# A structural equation model to explore sociodemographic, macroeconomic, and health factors affecting life expectancy in Oman

**DOI:** 10.11604/pamj.2022.41.75.28488

**Published:** 2022-01-26

**Authors:** Anak Agung Bagus Wirayuda, Sanjay Jaju, Yaqoub Alsaidi, Moon Fai Chan

**Affiliations:** 1 Department of Family Medicine and Public Health, Sultan Qaboos University, Al-Khoud, Oman

**Keywords:** Structural equation model, sociodemographic, macroeconomic, health-status and resources, life expectancy, Oman

## Abstract

**Introduction:**

the factors determining life expectancy (LE) are crucial for policymakers to study in implementing an effective and accurate intervention in society. In Oman, the available data over the past four decades were not extracted to develop a statistical model to understand how the sociodemographic (SD), macroeconomic (ME), and health-status and resources (HSR) factors affecting LE. The study was aimed at creating a representative model to explain the factors affecting LE in Oman and examine the direct and indirect effects of SD, ME, and HSR in LE in Oman.

**Methods:**

the research was a retrospective, ecological, time-series study design to collect the annual published data on SD, ME, and HSR in Oman from all available resources from 1978 to 2018. The data were then analyzed with structural equation modeling (SEM) method using IBM® SPSS® Amos 24 for the study of their impacts in LE.

**Results:**

in Oman, using SEM, the SD, ME, and HSR significantly and directly affected LE by the estimate of -0.92 (p < 0.001), -0.15 (p < 0.001), and 0.23 (p < 0.001) respectively.

**Conclusion:**

the study was the first attempt to analyze all the different aspects of LE comprehensively in Oman. In the case of Oman, the health resource is an important factor that need to be addressed to increase or to maintain the current LE. Hence, during social hardship or economic recession, health-related support by the government should be continued or even improved because of its positive effect on LE.

## Introduction

The worldwide life expectancy (LE) at birth in the 21^st^ century showed an uptrend pattern that resulted in the current average LE of more than 70 years old, which is higher than that in any country back in 1950 [1]. LE is one of the key metrics in public health performance [1, 2]. LE at birth provide the assessment of the mortality pattern among all age groups each year, including children, adolescents, adults, and the elderly [1]. The importance of LE has been emphasized in the United Nations Millennium Declaration in the year 2000, the past Millennium Development Goals (MDG) 2015, and the current Sustainable Development Goals (SDG) 2030 [3]. As in Gulf Cooperation Council (GCC) countries, consisting of Oman, Kuwait, Bahrain, Saudi Arabia, Qatar, and United Arab Emirates, the well-being and health of the populations have undergone significant changes due to the economic progress that brings improvement in fertility rates, infant mortality rates, and LE [4]. Oman's LE has increased substantially since the 1950´s when the Omani LE just lasted around 33 years on average. According to the World Health Organization (WHO), in 2018, the average LE in Oman was approximately 77.0 (Male 75.3, female 79.5), which placed Oman to be ranked the 45th worldwide [5, 6]. Dwindling oil revenues have significantly been used to help the average citizens' well-being through modernizations and human development, which were not seen before the renaissance of Oman. Since 1971, the development of health infrastructures, educational facilities, and economic improvements has majorly contributed to the rise of living standards, hence increasing LE of common citizens of Oman as they receive free basic health care, with a royal decree from the late HM Sultan Qaboos bin Said Al Said [5, 7]. Oman was ranked 8^th^ for their overall healthcare performance in the world in year 2000, but the position then declined significantly as the country was not included in the top 30 of nations with the most efficient health care in 2014 [8, 9]. The related ranking system observes the efficiency score of each country by measuring the change in LE compared with the change in the Gross Domestic Product (GDP) per capita and change in the health-care cost per capita [9, 10]. Based on those facts, there are room for improvements in Oman´s healthcare system and determining factors affecting LE in Oman has a crucial policy implication in rectifying the previous status in the global stage in performing a better overall healthcare performance in the years ahead, especially by addressing the sociodemographic and macroeconomic impact on its health output (LE).

Different researches have tried to identify the factors impacting LE in different countries, which comprise economic, demographic, and health factors [11-14]. Social factors such as the mean years of schooling, the total fertility rate, the number of physician density, and HIV prevalence had significant direct and indirect effects in LE [11]. Infant Mortality Rate (IMR) was negatively correlated to LE through health resources in Indonesia, the Philippines, and Vietnam [13]. Economic growth had a positive impact in LE in both low and high-income countries, mainly represented by GDP [13-15]. The employment ratio for both men and women also played an important role in LE, as the increase of unemployment might also decrease LE [13-15]. As in Oman, inflation and per capita income had a negative but insignificant relationship to LE [16]. This previous finding demands further study since it differs from the result found in other countries [11-14]. For health factors, the promotion of health standards and health infrastructures were directly related to LE of the people in both developing and developed countries [1].

The number of health service providers and health expenditures significantly correlated with LE [12, 13, 17-20]. In Oman, non-contagious and lifestyle-related diseases became substantially more prevalent within the past decade, especially obesity as almost 27 percent of Omani adults were obese in 2017, and Oman was ranked the 39th most obese country [21, 22]. On the other hand, obesity had a significant effect on mortality and life expectancy [23]. Another significant factor for health was air pollution which had a significant negative impact in LE [24]. Mental disorders and substance use also significantly reduced LE of the population [25,26]. Though the impact of sociodemographic, macroeconomic, and health factors in LE has been studied in many other countries, there has been little research conducted for LE´s determinants within the GCC region, especially Oman. The study was aimed to create a representative model to explain the factors affecting LE in Oman. In particular, this study examined the direct and indirect effects of sociodemographic (SD), macroeconomic (ME), and health-status and resources (HSR) in LE in Oman.

## Methods

***Design:*** the study was conducted as a retrospective, ecological, time-series study design to collect the annual data for Oman from all available resources from 1978 to 2018. The period under the study was chosen because before the early 70s there was a lack of official data available in Oman since the renaissance and the modernization of the Sultanate only started in 1970.

***Study area/setting:*** the study area is within the the Sultanate of Oman. The setting is based on the annual population data of the sultanate from 1978 to 2018.

***Data sources and ethical approval:*** the study used published data provided from the Ministry of Health of Oman, the Center for Statistics and Information of Oman, the World Health Organization, the World Bank, and other databases which were available in the public domain. Due to the utilization of secondary data, the ethical exemption was obtained from the IRB of Sultan Qaboos University (MERC #2032). The data collection started from December 2019 to December 2020. The data were evaluated for its sources and accuracy, mainly by deleting items with large numbers of missing data. Although there was no clear guideline on how large the baseline for the percentage of the missing data is allowed, this study deleted any items with more than 20% missing data [27]. If any data had been missing at random, it would have been replaced by the multiple imputation method [28].

### The model and variables

**Figure 1:**
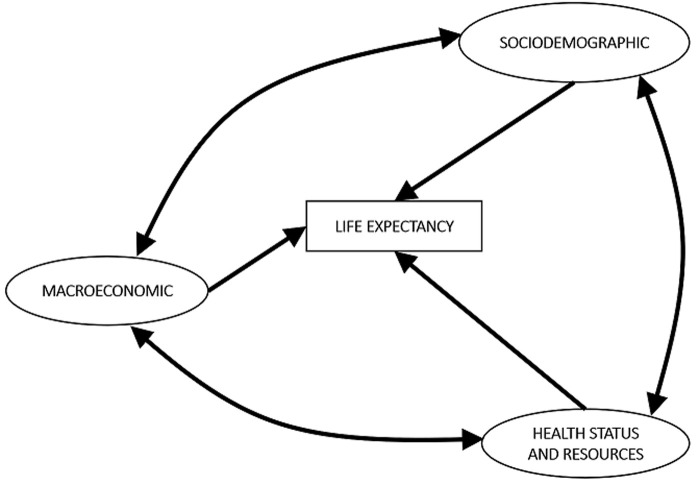
the conceptual model of sociodemographic, macroeconomic, and health status and resources effect on life expectancy in Oman (1978-2018)

A conceptual model was used based on previous studies conducted in the ASEAN countries (Figure 1) [12-14]. Items symbolized with the rectangular shape represent the observable variables, while the oval-shaped items represent the latent (non-observable) variables. An arrow indicates the direction of a hypothesized predictive or regression relationship, while the bidirectional arrow implies correlation or covariance between two variables. All the observable items included in this study have undergone the data cleaning process and shown a certain level of impact in LE from the previous related studies [11-17, 29, 30]. LE is the dependent variable, defined as an average number of years of life remaining at a given age, particularly at birth, from which the calculation is based on the analysis of life tables [1]. There are three main latent variables (SD, ME, HSR) expected to impact LE. SD consists of 5 observable variables: the primary and secondary school enrollment as the percentage of primary/secondary school enrollment annually [31, 32], IMR as the rate of infant mortality per 1000 live births annually [33], fertility rate as the total birth per woman annually [34], and female adult mortality rate as the ratio of adult mortality of female population annually [35]. In the ME, observable variables include the GDP per capita as the gross domestic product per number of population in current US$ annually [11-15], the dependency ratio as the percentage of the working-age population annually [36], and capital investment in billion US$ annually [37]. For the HSR, observable variables include CO2 emissions as the carbon dioxide emitted in metric tons per capita annually [24], mental and substance use disorders as the percentage of mental disorders and substance use within a share of total disease burden annually [38], and the prevalence of obesity for females or males as the percentage of adult female/male population with obesity in the given year [39] (Figure 1).

**Table 1:** the correlation matrix of variables of sociodemographic, macroeconomic, and health status and resources effect on life expectancy in Oman (1978-2018)

	LE	PSE	SSE	IMR	FR	AMR (f)	GDPpc	DR	CI	CO2 E	MSU	O(f)	O(m)
LE	1												
PSE	.99**	1											
SSE	.99**	.98**	1										
IMR	.99**	-.98**	-.99**	1									
FR	-.97**	-.93**	-.98**	.98**	1								
AMR(f)	-.48**	-.39*	-.54**	.47**	.60**	1							
GDPpc	.81**	.79**	.81**	-.78**	-.79**	-.69**	1						
DR	-.93**	-.90**	-.95**	.92**	.94**	.73**	-.91**	1					
CI	.77**	.73**	.78**	-.72**	-.73**	-.73**	.93**	-.90**	1				
CO2E	.80**	.75**	.81**	-.78**	-.82**	-.69**	.88**	-.88**	.82**	1			
MSU	.51**	.48**	.52**	-.50**	-.53**	-.50**	.63**	-.61**	.63**	.60**	1		
O(f)	.78**	.81**	.78**	-.79**	-.77**	-.40**	.70**	-.75**	.65**	.54**	.45**	1	
O(m)	.96**	.92**	.96**	-.93**	-.93**	-.64**	.86**	-.97**	.87**	.89**	.57**	.69**	1

LE: Life expectancy at birth. PSE: Primary school enrollment. SSE: Secondary school enrollment. IMR: Infant mortality rate. FR: Fertility rate. AMR(f): Adult mortality rate (female). GDPpc: GDP per capita. DR: Dependency ratio. CI: Capital investment. CO2 E: CO2 emission. MSU: Mental and substance use disorders. O(f): Prevalence of obesity (female). O(m): Prevalence of obesity (male). *. Correlation is significant at the 0.05 level; **. Correlation is significant at the 0.01 level

### Statistical analysis

The study utilized Structural Equation Modeling (SEM) to measure the direct and indirect effect according to the proposed model, using IBM® SPSS® Amos 24 as the statistical tool. The relations among variables in the SEM were defined by a series of equations that described hypothesized structures of relationships. An asymptotic correlation matrix was used to estimate the model parameters (Table 1). Once a model was established, absolute measures and incremental fit indices were used to examine its goodness of fit [38, 39]. In the absolute measures, the normed χ^2^ (=χ^2^/df) with cut-off < 4.0 indicates a good fit [38]. Tucker-Lewis Index (TLI) ranges from 0 to 1, of which at least 0.90 value is accepted as a good fit [40, 41]. Root Mean Square Error of Approximation (RMSEA) ranging from 0 to 1 indicates a well-fit with below 0.05 [40, 41]. Instead of using the RMSEA, the Standardized Root Mean Square Residual (SRMR) is recommended if the sample or degree of freedom is < 50 [40, 41]. SRMR, which ranges from 0 to 1, indicates a well fit when the value is below 0.08 [42, 43]. The Akaike´s Information Criterion (AIC), Browne-Cudeck Criterion (BCC), Consistent version of the AIC (CAIC), and Expected Cross-Validation Index (ECVI) compare two or more models of which LEast values reflect the better model [40,41]. The Incremental Fit Indices, which differ from the absolute measures, compare a given model to an alternative, usually the conceptual model. They consist of the Incremental Fit Index (IFI), Normed Fit Index (NFI), and Comparative Fit Index (CFI), which range from 0 to 1, and the threshold is at least 0.90 [40, 41]. If the model had not been adequate, modifications by removing those not significant relationships among variables would have been performed until a model reached a good fit. AMOS program was used to perform all the analysis, and all tests were set at a 5% level of significance (Table 1).

**Data availability:** the data used in this study will be made available with reasonable request to the corresponding author.

**Funding:** this study was funded by the Deanship Research Grant of Sultan Qaboos University (RF/MED/ FAMCO/19/03).

**Ethical approval:** an ethical exemption was obtained from the IRB of Sultan Qaboos University (MERC #2032).

## Results

**Figure 2:**
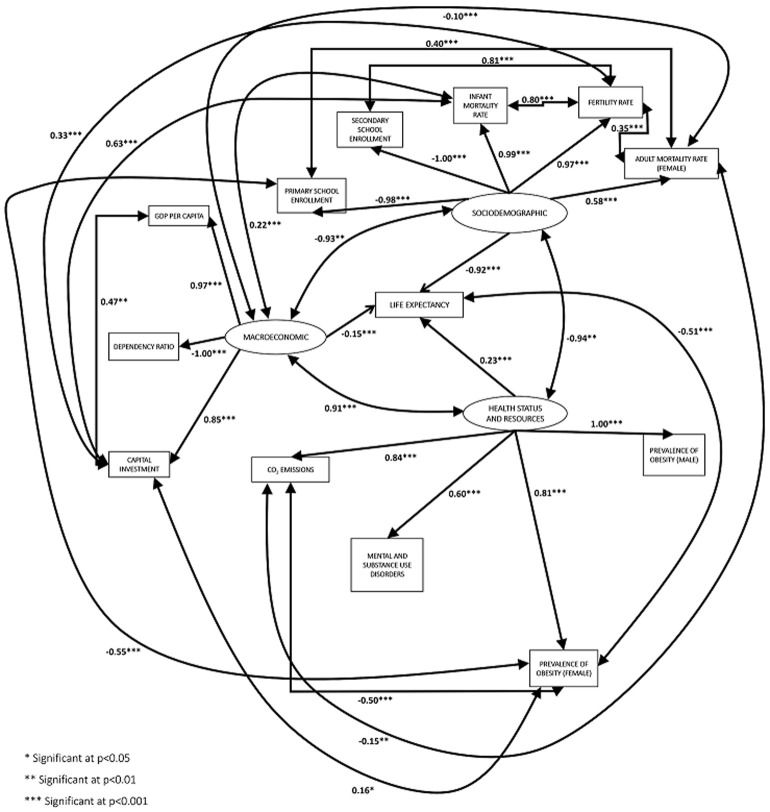
the final model of sociodemographic, macroeconomic, and health status and resources effect on life expectancy in Oman (1978-2018)

In Table 2, the initial conceptual model resulted in poor fit on all indices (*χ^2^/df*=7.5, TLI=0.6, RMSEA=0.4, SRMR=0.08, AIC=514.9, IFI=0.7, NFI=0.7, CFI=0.7), it needed further modifications to improve the goodness-of-it. There was a significant improvement, as shown from model 1 to model 3. The model fit indices showed that, overall, model 3 (*χ^2^/df*=3.3, TLI=0.9, RMSEA=0.2, SRMR=0.06, AIC=234.4, IFI=0.9, NFI=0.9, CFI=0.9) had better results than the other 3 models. Using standardized estimates, based on model 3 (Figure 2), the SD factor significantly and directly affected LE by -0.92 (p < 0.001), the ME factor significantly and directly affected LE by -0.15 (p < 0.001), and the HSR significantly and directly affected LE by 0.23 (p < 0.001). No indirect effect resulted. Thus, the analysis of the model proved that there were significant direct effects of SD, ME, and HSR on LE in Oman (Table 2, Figure 2).

**Table 2:** comparison of goodness-of-fit indices on models for life expectancy in Oman (1978-2018)

	Conceptual Model	Model 1	Model 2	Model 3	Cut-off value
Absolute Measures	df=60	df=46	df=43	df=41	
χ2/df	7.5	5.9	4.3	3.3	< 4.0
TLI	0.6	0.7	0.8	0.9	≥ 0.9
SRMR	0.08	0.06	0.07	0.06	< 0.08
RMSEA	0.4	0.4	0.3	0.2	< 0.05
AIC	514.9	362.1	280.4	234.4	the smaller the better
BCC	548.3	410.6	332.1	288.2	the smaller the better
CAIC	599.0	484.2	410.7	370.1	the smaller the better
ECVI	12.9	9.1	7.0	5.9	the smaller the better
Incremental Fit Index					
IFI	0.7	0.9	0.9	0.9	≥ 0.9
NFI	0.7	0.8	0.9	0.9	≥ 0.9
CFI	0.7	0.8	0.9	0.9	≥ 0.9

TLI: Tucker-Lewis Index. SRMR: Standardized Root Mean Square Residual. RMSEA: Root mean square error of approximation. AIC: Akaike´s Information Criterion. BCC: Browne-Cudeck Criterion . CAIC: Consistent version of the AIC. ECVI: Expected Cross-Validation Index. IFI: Incremental Fit Index. NFI: Normed Fit Index . CFI: Comparative Fit Index.

## Discussion

The result showed that SD and ME had a significant and negative effect in LE while HSR had a significant and positive effect in LE. The study is the first attempt to compile all different aspects of LE comprehensively in Oman. A potential explanation of the negative effect of SD in LE can be seen from the education factor, in particular the lifestyle of people with higher education that include: more stress due to more complex responsibilities at work, bad diet, long working hours, and less physical activity which, in the long run, may affect LE in general [44]. This study also showed that economic growth does not always correlate positively with LE, as some other studies showed the same [45, 46]. In South Africa, LE had a downturn from 1980 to 2012 despite the increase of GDP by sevenfold [45]. As in high-income countries, the relationship can go into an inverse or negative direction as the economic inequalities also get wider and wealthy people also have a tendency to follow an unhealthy habit such as smoking and a poor diet [45-48]. As for the HSR factor, the result of this study goes in line with the other studies from other regions as the most important factor that increases LE over the past decades is the progress of health care among different health care systems [12-14, 18, 19].

ME and SD status are a complex concept that may affect health directly or indirecly through associated functions as one aspect include income, educations, wealth, whereas the other also include status, as the position or hierarchy within the particular social class [44]. In general, the ME factors have a powerful impact on LE as rising inflation decreases purchasing power of the households, as for every one percent increase in inflation in Oman, the LE decreases by approximately 0.005 percent [17]. In comparison to Italia, there was a significantly negative association between income inequalities, as regions with higher income inequality within the population had a significantly lower LE than regions where the populations have comparatively lowed income inequality [49]. This particular inequality might be the case of negative ME impact on LE in the population of Oman, as this study is an ecological design with population aggregate while higher income is commonly associated with greater longevity or LE throughout the income distribution [49, 50].

The study findings may be used by the Ministry of Health and the policymakers for Oman's benefit, especially to help achieve the Health Vision 2050 of Oman as LE belongs to one of the important indicators of overall healthcare performance. The result showed that HSR was the only significant and positive effect in LE. Hence, the support from the government for healthcare intervention will always be needed despite the negative SD and ME effect. Moreover, from a policy perspective, such an understanding can help improve the efficiency of health system financing through an improved dynamic allocation of medical resources across age groups, mitigating purely demographic pressures.

As the study used an ecological design, confounding may occur in the population level, because the data does not address the sub-group that may differ in the average aggregate level [51]. The study might also have limited observable variables to be used in the SEM since there were a lot of missing data and lack of information from different periods. This might affect the RMSEA value of the final chosen model (model 3), with a high value (=0.2) indicating a poor-fit. However, this result was perhaps obtained due to a small sample (df) that RMSEA may indicate a poor fitting model in most of the cases of small sample size [52]. Similar studies in the ASEAN region may provide more indicators or items and better model fit indices because of the abundance of data provided, albeit the sample size was smaller than this study in Oman [12-14]. In the future, a further study in Oman may be needed to accomplish a perfect model with more data collected over time. The ASEAN studies also provided indirect effects, mainly through the health and demographic determinants, while there were no indirect effects detected in the final model of this study [12-14].

## Conclusion

The study conducted in Oman provides a model to explain the relationship between SD, ME, and HSR with LE from 1978 to 2018. The result showed significant direct effects of all the main determinants. In Oman´s case, the study implied that healthcare resources are important factors that need to be addressed to increase or maintain the current LE. Thus, during social hardship or economic recession, health-related support by the government should be continued or even improved because of its positive effect on LE. The model showed that SD, ME, and HSR have significant correlations among themselves, which proves that each factor should be put into account in the decision-making or intervention. Hence, health literacy and a socio-cultural approach may be needed to educate a better lifestyle, as obesity and other non-infectious diseases are prevalent in Oman. Some of the health status had a significant impact on the model, such as obesities, and mental and substance use disorder. The model also showed that each disease and health status also correlate with many other indicators, such as male obesity and primary school enrollment. This may imply that controlling lifestyle-related disease burden in Oman may improve the entire model. Thus, the promotive and preventive effort to combat obesity campaigned by the health workers and the primary health care in the society may be needed even more. It is also important to explore mental health literacy within the society to give a better insight into the promotive and preventive campaign to solve mental health disorders and substance abuses. In the form of CO2, pollution also made a significant contribution to HSR, and this finding can be referred to the industrial investment that adheres to the environmental standard. The study may also open up possible future research for more comprehensive research for other countries with similar demographics and economic characteristics. With SEM, it is possible to determine whether the same theoretical framework or model works equally well for explaining LE data in other countries with similar demographics characteristics, such as the countries in the Middle East and North Africa.

### What is known about this topic

- *Life expectancy is affected by sociodemographic, macroeconomic, and health-related factors worldwide;*
- *Sociodemographic, macroeconomic, and health-related factors may correlate with each other;*
- *The determinants of life expectancy may perform differently between low-income and high-income countries*.

### What this study adds

- *Oman´s sociodemographic and macroeconomic determinants have a negative effect on life expectancy;*
- *During the economic recession and social calamity, health-related service and support should be continued or even improved despite the hardship because the health status and resources of Oman is the only main determinant that performs a positive effect on life expectancy;*
- *By using a similar approach, it is possible to determine whether the same theoretical model works equally well for explaining LE data in other countries with similar demographics characteristics, such as the countries in the Middle East and North Africa*.

## References

[1] Roser M, Ortiz-Ospina E, Ritchie H (2013). Life expectancy. Our World in Data.

[2] Ritchie H (2019). 12 key metrics to understand the state of the world. Our World in Data.

[3] World Health Organization (WHO) (2015). From MDGS to SDGS: General Introduction.

[4] Khoja T, Rawaf S, Qidwai W, Rawaf D, Nanji K, Hamad A (2017). Health Care in Gulf Cooperation Council Countries: A Review of Challenges and Opportunities. Cureus.

[5] World Health Organization (2018). World Health statistics 2018: monitoring health for the SDGs.

[6] Macrotrends (2021). Oman life expectancy 1950-2021. Macrotrends.

[7] Al Riyami A (2012). Health vision 2050 Oman: A committed step towards reforms. Oman Med J.

[8] Tandon A, Murray C, Lauer J, Evans D (2000). Measuring overall health system performance for 191 countries.

[9] Bloomberg Rankings (2014). Most efficient health care. Bloomberg.

[10] Schütte S, Acevedo P, Flahault A (2018). Health systems around the world-a comparison of existing health system rankings. Journal of global health.

[11] Mondal MNI, Shitan M (2014). Relative importance of demographic, socioeconomic and health factors on life expectancy in low-and lower-middle-income countries. J Epidemiol.

[12] Chan MF, Taylor BJ (2013). Impact of demographic change, socioeconomics, and health care resources on life expectancy in Cambodia, Laos, and Myanmar. Public Health Nurs.

[13] Chan MF (2015). The impact of healthcare resources, socioeconomic status, and demographics on life expectancy: A cross-country study in three Southeast Asian countries. Asia-Pacific J Public Heal.

[14] Chan MF, Kamala DM (2015). Factors affecting life expectancy: Evidence from 1980-2009 data in Singapore, Malaysia, and Thailand. Asia-Pacific J Public Heal.

[15] Austin KF, Mckinney LA (2012). Disease, war, hunger, and deprivation: A cross-national investigation of the determinants of life expectancy in less-developed and sub-saharan african nations. Sociol Perspect.

[16] Ketenci N, Murthy VNR (2018). Some determinants of life expectancy in the United States: results from cointegration tests under structural breaks. J Econ Financ.

[17] Ali A, Ahmad K (2014). The Impact of Socio-Economic Factors on Life Expectancy in Sultanate of Oman?: An Empirical Analysis. Middle-East J Sci Res.

[18] Asandului L, Roman M, Fatulescu P (2014). The efficiency of healthcare systems in Europe: A data envelopment analysis approach. Procedia Econ Financ.

[19] Amiri A, Solankallio-Vahteri T (2019). Nurse staffing and life expectancy at birth and at 65 years old: Evidence from 35 OECD countries. Int J Nurs Sci.

[20] Nixon J, Ulmann P (2006). The relationship between health care expenditure and health outcomes: Evidence and caveats for a causal link. Eur J Heal Econ.

[21] Thelwell K (2019). 10 Facts About Life Expectancy in Oman. The Borgen Project.

[22] Global Obesity Levels Obesity.

[23] Peeters A, Barendregt JJ, Willekens F, Mackenbach JP, Al Mamun A, Bonneux L (2003). Obesity in adulthood and its consequences for life expectancy: a life-table analysis. Ann Intern Med.

[24] Amuka JI, Asogwa FO, Ugwuanyi RO, Omeje AN (2018). Climate change and Life Expectancy in a Developing Country: Evidence from Greenhouse Gas (CO2) Emission in Nigeria. Int J Econ Financ Issues.

[25] Ilyas A, Chesney E, Patel R (2017). Improving life expectancy in people with serious mental illness: should we place more emphasis on primary prevention?. Br J Psychiatry.

[26] McGrath JJ, Lim CCW, Plana-Ripoll O, Holtz Y, Agerbo E, Momen NC (2020). Comorbidity within mental disorders: a comprehensive analysis based on 145 990 survey respondents from 27 countries. Epidemiol Psychiatr Sci.

[27] Dong Y, Peng C-YJ (2013). Principled missing data methods for researchers. Springerplus.

[28] Byrne BM (2016). Structural Equation Modeling With AMOS.

[29] Bayati M, Akbarian R, Kavosi Z (2013). Determinants of life expectancy in eastern Mediterranean region: A health production function. Int J Heal Policy Manag.

[30] Duque AM, Peixoto MV, Lima SVMA, Goes MAO, Santos AD, Araújo KCGM (2018). Analysis of the relationship between life expectancy and social determinants in a North-Eastern region of Brazil, 2010-2017. Geospat Health.

[31] Baluch MUH, Shahid S (2008). Determinants of Enrollment in Primary Education: A Case Study of District Lahore. Pak Econ Soc Rev.

[32] Baker DP, Halabi S, Michalos AC (2014). School Enrollment. Encyclopedia of Quality of Life and Well-Being Research.

[33] Blaxter M (1982). The health of the children: a review of research on the place of health in cycles of disadvantage. J R Coll Gen Pract.

[34] Jensen MB, Priskorn L, Jensen TK, Juul A, Skakkebaek NE (2015). Temporal Trends in Fertility Rates: A Nationwide Registry Based Study from 1901 to 2014. PLOS ONE.

[35] Bradshaw D, Timaeus IM, Jamison DT, Feachem RG, Makgoba MW, Bos ER, Baingana FK, Hofman KJ (2006). Levels and Trends of Adult Mortality. Disease and Mortality in Sub-Saharan Africa.

[36] Wachs D, Roman-Urrestarazu A, Brayne C, Onrubia-Fernández J (2020). Dependency ratios in healthy ageing. BMJ Global Health.

[37] Currie J, Goodman J, Bradley S, Green C (2020). Chapter 18 - Parental socioeconomic status, child health, and human capital. The Economics of Education (Second Edition).

[38] Mclellan AT (2017). Substance Misuse and Substance use Disorders: Why do they Matter in Healthcare?. Trans Am Clin Climatol Assoc.

[39] Flegal KM, Graubard BI, Williamson DF, Gail MH (2015). Underweight, Overweight, and Obesity. JAMA.

[40] Kline RB (2011). Principles and practice of structural equation modeling.

[41] Hooper D, Coughlan J, Mullen M (2008). Structural Equation Modelling: Guidelines for Determining Model Fit. Electron J Bus Res.

[42] Chen F, Curran PJ, Bollen KA, Kirby J, Paxton P (2008). An empirical evaluation of the use of fixed cutoff points in RMSEA test statistic in structural equation models. Sociol Methods Res.

[43] Kenny DA, Kaniskan B, McCoach DB (2015). The Performance of RMSEA in Models With Small Degrees of Freedom. Sociol Methods Res.

[44] Bilas V, Franc S, Bošnjak M (2014). Determinant factors of life expectancy at birth in the european union countries. Coll Antropol.

[45] Biciunaite A (2014). Economic growth and life expectancy - do wealthier countries live longer?. Euromonitor International.

[46] Preston SH (2007). The changing relation between mortality and level of economic development. Int J Epidemiol.

[47] Bacci ML (2017). A Concise History of World Population. John Wiley & Sons.

[48] Messias E (2003). Income Inequality, Illiteracy Rate, and Life Expectancy in Brazil. Am J Public Health.

[49] De Vogli R, Mistry R, Gnesotto R, Cornia G (2005). Has the relation between income inequality and life expectancy disappeared? Evidence from Italy and top industrialised countries. Journal of Epidemiology & Community Health.

[50] Chetty R, Stepner M, Abraham S, Lin S, Scuderi B, Turner N (2016). The Association Between Income and Life Expectancy in the United States, 2001-2014. JAMA.

[51] Salkeld D, Antolin M (2020). Ecological fallacy and aggregated data: a case study of fried chicken restaurants, obesity and Lyme disease. Ecohealth.

[52] Kenny D, Kaniskan B, McCoach D (2015). The performance of RMSEA in models with small degrees of freedom. Sociological methods & research.

